# Accelerated Metabolite Levels of Aerobic Glycolysis and the Pentose Phosphate Pathway Are Required for Efficient Replication of Infectious Spleen and Kidney Necrosis Virus in Chinese Perch Brain Cells

**DOI:** 10.3390/biom9090440

**Published:** 2019-09-02

**Authors:** Xixi Guo, Shiwei Wu, Ningqiu Li, Qiang Lin, Lihui Liu, Hongru Liang, Yinjie Niu, Zhibin Huang, Xiaozhe Fu

**Affiliations:** 1 Pearl River Fisheries Research Institute, Chinese Academy of Fishery Sciences, Key Laboratory of fishery Drug Development, Ministry of Agriculture and Rural Affairs, Key Laboratory of Aquatic Animal Immune Technology, Guangdong Provinces, Guangzhou 510380, China; 2 College of Fisheries and Life Science, Shanghai Ocean University, Shanghai 201306, China

**Keywords:** ISKNV, *Siniperca chuatsi*, glucose metabolism reprogramming, aerobic glycolysis, pentose phosphate pathway (PPP), TCA cycle

## Abstract

Glucose is a main carbon and energy source for virus proliferation and is usually involved in the glycolysis, pentose phosphate pathway (PPP), and tricarboxylic acid cycle (TCA cycle) pathways. In this study, we investigated the roles of glucose-related metabolic pathways during the replication of infectious spleen and kidney necrosis virus (ISKNV), which has caused serious economic losses in the cultured Chinese perch (*Siniperca chuatsi*) industry. We found that ISKNV infection enhanced the metabolic pathways of the PPP and the TCA cycle at the early stage of the ISKNV infection cycle and enhanced the glycolysis pathway at the late stage of the ISKNV infection cycle though the comprehensive analysis of transcriptomics, proteomics, and metabolomics. The advanced results proved that ISKNV replication induced upregulation of aerobic glycolysis at the late stage of ISKNV infection cycle and aerobic glycolysis were required for ISKNV multiplication. In addition, the PPP, providing nucleotide biosynthesis, was also required for ISKNV multiplication. However, the TCA cycle involving glucose was not important and necessary for ISKNV multiplication. The results reported here provide new insights into viral pathogenesis mechanism of metabolic shift, as well as antiviral treatment strategies.

## 1. Introduction

Glucose is a main carbon and energy source for cell and virus proliferation. Usually, glucose is involved in the glycolysis, pentose phosphate pathway (PPP), and tricarboxylic acid cycle (TCA cycle) pathways. Normally, glucose is considered the main contributor of cellular adenosine triphosphate (ATP) through its oxidative phosphorylation via the TCA cycle [1]. The Warburg effect (or aerobic glycolysis) is an abnormal glycolysis response that is associated with cancer cells to support their high energy requirements and high rates of macromolecular synthesis. In cancer cells, the main hallmark of the Warburg effect is aerobic glycolysis, in which glucose consumption and lactate production are both increased, even in the presence of oxygen [2,3]. This metabolic reprogramming was also elicited by some viruses, such as human papillomavirus (HPV) [4], human cytomegalovirus (HCMV) [5,6], white spot syndrome virus (WSSV) [7,8], Kaposi’s sarcoma herpesvirus (KSHV) [9], and hepatitis C virus (HCV) [10]. The Warburg effect, along with the PPP and fatty acid metabolism, are used to support viral energy requirements [2,11]. In our previous study, we also found that the ISKNV infection promoted the enzymes expression of glucose metabolism [12].

Since the early 1990s, infectious spleen and kidney necrosis virus (ISKNV) has caused high mortality and serious economic losses in the Chinese perch (*Siniperca chuatsi*) farming industry. ISKNV is a large unique dsDNA virus, which contains a single linear dsDNA molecule comprised of 111,362 bp in length and 124 potential open reading frames (ORFs) [13]. It takes 72 h to complete one multiplication cycle in vitro, and during its lifecycle, the temporal expression of viral genes was found, including the immediate early genes ORF023 and ORF038, the early genes ORF034 and ORF093, and the late gene ORF006 (major capsid protein gene, MCP) [12]. In addition, ISKNV can infect more than 50 marine and freshwater fish species, including the species in the *Perciformes*, *Pleuronectiformes*, *Clupeiformes*, *Tetraodontiformes*, *Myctophiformes*, and *Mugiliformes* orders [14]. Due to its high incidence and serious influence on fish aquaculture industry, ISKNV has been listed by the International Epizootic Office (OIE) [15,16]. We previously reported that ISKNV infection induced glutaminolysis to accommodate the biosynthetic and energy needs for its efficient virus replication [17] and to promote the enzymes expression of glucose metabolism [18].

We found that the color of ISKNV-infected cell medium was changed from red to yellow rapidly, which led us to question whether ISKNV infection induced the glucose metabolic reprogramming. In present study, to determine the roles of glucose-related metabolic pathways during ISKNV multiplication, the global metabolic changes were investigated by transcriptomics, proteomics, and metabolomics analysis. The roles of glycolysis, the PPP, and TCA cycle were determined in ISKNV-infected CPB cells.

## 2. Materials and Methods

### 2.1. Cells and Virus

The Chinese perch brain cell line (CPB) was established in our lab [16] and maintained at 28 °C in Leibovitz’s L-15 medium (GIBCO, New York, NY, USA) supplemented with 10% FBS (GIBCO, New York, NY, USA). ISKNV-QY was isolated and stored in our laboratory [16]. The virus was propagated in CPB cells at 28 °C and the viral titer was determined by a TCID_50_ assay.

### 2.2. Antibodies and Pharmaceuticals

Dehydroepaimdrosterone (DHEA), 2-Deoxy-d-glucose (2-DG), Dichloroacetate (DCA), and dimethyl sulfoxide (DMSO) were purchased from Sigma-Aldrich (St. Louis, MO, USA). DHEA and DCA were dissolved in DMSO to a stock concentration of 100 μM/L, and 2-DG was dissolved in H2O to a stock concentration of 10 mg/mL. These reagents need to be diluted to a working concentration using L-15 medium just before use. The rabbit polyclonal antibodies against MCP were prepared and stored in our laboratory. The rabbit polyclonal antibodies against glucose-6-phosphate dehydrogenase (G6PDH), hexokinase 1 (HK1), glucose-6-phosphate isomerase (GPI), enolase (ENO), pyruvate kinase (PK), lactate dehydrogenase (LDH), and pyruvate dehydrogenase complex (PDHx), as well as the mouse monoclonal antibody against β-actin, were purchased from Proteintech (USA). The secondary antibody was purchased from KPL (USA).

### 2.3. Cell Viability Assay

The CPB cells viability was detected in complete medium treated with 2-DG (0, 0.05, 0.1, 0.2, 0.3, 0.4 mg/mL), DHEA (0, 0.5, 1.0, 2.5, 5.0, 10 µM), or DCA (0, 0.5, 1.0, 2.5, 5.0, 10 µM) using the CellTiter 96^®^ AQ_ueous_ One Solution Cell Proliferation Assay (MTS) (Promega, Madison, WI, USA) according to the manufacturer’s instructions. A medium without 2-DG, DHEA, or DCA was used as a control.

### 2.4. Quantification of ISKNV Copies

CPB cells were pretreated with DHEA, 2-DG, or DCA for 4 h and then infected with ISKNV (MOI of 1.0). The total DNA of cell samples and supernatants infected with ISKNV at 36 and 48 h post-infection (hpi) was extracted with E.Z.N.A.^TM^ Tissue DNA Kit (OMEGA, Norcross, GA, USA) according to the manufacturer’s instructions. The viral copy number was determined by real-time PCR developed in our lab as described previously [19].

### 2.5. Transcriptomics and Proteomics Profile Analyses of CPB Cells Infected with ISKNV

Transcriptomics and iTRAQ labeling quantitative proteomics profile analysis were described in full detail in our previous study [12,18].

### 2.6. Metabolomics Profile Analysis of CPB Cells Infected with ISKNV by Liquid Chromatography Mass Spectrometry (LC-MS)

At 24 and 72 hpi, six parallel samples (three replicate samples were pooled for one parallel sample) of ISKNV-infected or non-infected CPB cells were collected. Metabolic profiling was performed on an Agilent 1290 Infinity LC system (Agilent Technologies, Santa Clara, CA, USA) coupled with an AB SCIEX Triple TOF 6600 System (AB SCIEX, Framingham, MA, USA) [20]. The analysis process was conducted with the assistance of Applied Protein Technology Co., Ltd. (Shanghai, China).

### 2.7. Gene Transcription of Glucose Metabolism Pathway during ISKNV Multiplication

The transcriptional levels of Hexokinase 1 (HK1), glucose-6-phosphate isomerase (GPI), glucose-6-phosphate dehydrogenase (G6PDH), enolase (ENO), pyruvate kinase (PK), lactate dehydrogenase (LDH), and pyruvate dehydrogenase kinase (PDK) genes were evaluated by quantitative reverse-transcription PCR (qRT-PCR). The primers are listed in Table 1. Total RNAs of ISKNV-infected or non-infected CPB cells (three parallel samples) were isolated at 24 and 72 hpi with TRIzol reagent (Invitrogen, Carlsbad, CA, USA). The amount of 1 mg RNA was used for cDNA synthesis using Abm 5×All-In-One RT MasterMix Kit (Abm, Guangzhou, China). qRT-PCR was performed with a SYBR^®^ Premix Ex Taq™kit (Takara, Japan) on an ABI 7500 machine (Applied Biosystems, Foster City, CA, USA), and 18S rRNA was used as the internal control as described previously [21]. The cycling parameters were 95 °C for 5 min, followed by 40 cycles of 95 °C for 10 s and 60 °C for 30 s. All reactions were done in triplicate and dissociation curve analysis was performed after each assay to determine target specificity. The relative expression ratio of the target genes versus the 18S rRNA gene was calculated using the 2^−∆∆CT^ method. All data was given in terms of relative mRNA expression, expressed as the mean ± the standard deviation (SD) of the mean [22].

### 2.8. Determination of Glucose Consumption and Lactate Accumulation in Supernatants

Supernatants of ISKNV-infected cells at different time points (24, 36, 48, and 60 hpi) were harvested. Glucose concentration was measured by EnzyChrom™ Glucose Assay Kit (YEASEN, Shanghai, China). Lactate accumulation was measured by EnzyChrom^TM^ L-Lactate Assay Kit (YEASEN, Shanghai, China) according to the manufacturer’s instructions, together with a selectra-XL chemical analyzer (Huizen, The Netherlands).

### 2.9. CPB Cell Energy Phenotype Measurement Post ISKNV Infection

To assess CPB cell energy phenotype alteration post-ISKNV infection, the oxygen consumption rate (OCR) and extracellular acidification rate (ECAR) of ISKNV-infected cells and control cells were determined using Seahorse XFp Cell Energy Phenotype Test Kit according to manufacturer’s protocol, together with Seahorse XFp Analyzer (Agilent, Santa Clara, CA, USA). At 60 hpi, control and ISKNV-infected CPB cells at equal densities (4 × 10^4^ cells per well) were seeded in XFp Cell Culture Miniplate with 80 μL complete DMEM medium containing 2% FBS at 28 °C with 5% CO_2_ overnight. Then, the cell medium was replaced with seahorse XF base medium containing 10 mM Glucose, 1.0 mM Sodium pyruvate and 2 mM Glutamine, and the miniplate was placed in a CO_2_-free incubator at 37 °C for 1 h. After incubation, drugs were injected to the final concentration as 50 μM of oligomycin and 50 μM of carbonyl cyanide-4-(trifluoromethoxy)phenylhydrazone (FCCP) and the miniplate was transferred to the seahorse XFp extracellular flux analyzer (Agilent, Santa Clara, CA, USA). The OCR and ECAR were measured and normalized by cell number.

### 2.10. Western Blot Analysis

Samples were collected and lysed in RIPA buffer (with 1% PMSF). Proteins were separated by 12% SDS-PAGE, and then transferred onto Immobilon-P polyvinylidene difluoride membranes (Millipore, MA, USA). The membranes were blocked using 5% skimmed milk for 3 h at room temperature. Then, membranes were incubated with the indicated primary antibody (anti-HK1 (1:1000), anti-G6PDH (1:1000), anti-GPI (1:1000), anti-ENO1 (1:1000), anti-PK (1:1000), anti-LDH (1:1000), anti-PDHx (1:1000)), and subsequently incubated with secondary antibodies (1:5000). The image was visualized by chemiluminescence using Thermo Scientific Pierce Western Blot ECL Plus (Thermo, Waltham, MA, USA).

### 2.11. Statistical Analysis

Results are expressed as the means ± standard deviation (SD) from at least three experiments. Statistical differences between groups were determined by one-way analysis of variance (ANOVA). All tests were conducted using SPSS software (version 21.0, IBM, New York, NA, USA). Values were considered statistically significant at *p* < 0.05 and extremely significant at *p* < 0.01.

## 3. Results

### 3.1. ISKNV Infection Altered Glucose Metabolism in CPB Cells

**Figure 1:**
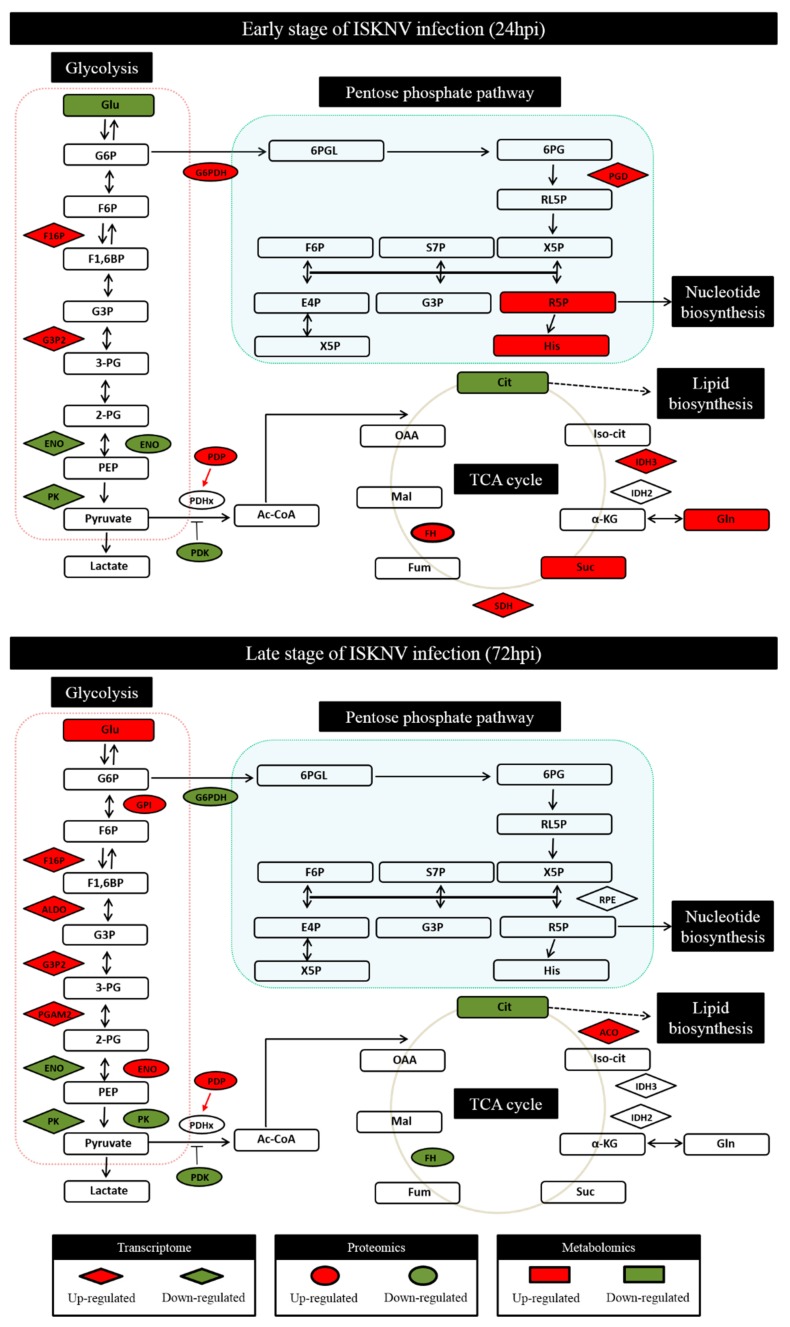
The infectious spleen and kidney necrosis virus (ISKNV) induced the changes of glucose metabolism in the cellular transcriptomic, proteomic, and metabolomics of Chinese perch brain cell line (CPB) cells at its replication cycle. Changes in the levels of enzymes of genes (rhombus), proteins (ellipses), and metabolites (rectangles) relative to control controls are color-coded to represent upregulation (red) or downregulation (green). Colorless boxes indicate that no difference was detected. Transcriptome date were collected from three groups of parallel samples of CPB cells using RNA-Seq. Proteomics date were collected from three groups of parallel samples of CPB cells using iTRAQ proteomics. Metabolomics data were collected from six groups of parallel samples of CPB cells using LC-MS. Numeric values for the transcriptomic and proteomic data are given in Hu et al. [18] and Wu et al. [12]. Abbreviations: Glucose (Glu), Glucose-6-phosphate (G6P), Fructose-6-phosphate (F6P), Fructose 1,6-bisphosphate (F1,6BP), Glyceraldehyde 3-phosphate (G3P); 3-phosphoglycerate (3-PG), 2-phosphoglycerate (2-PG), Phosphoenolpyruvate (PEP), Pyruvate, Acetyl-CoA (Ac-CoA), Glutamine (Gln), α-Ketoglutaric acid (α-KG), Succinate (Suc), Fumarate (Fum), Malate (Mal), Oxaloacetate (OAA), Citrate (Cit), Isocitrate (Iso-cit), Histidine (His), Xylulose 5-phosphate (X5P), Ribose 5-phosphate (R5P), Erythrose 4-phosphate (E4P), Sedoheptulose 7-phosphate (S7P), Ribulose 5-phosphate (RL5P), 6-Phospho-gluconate (6PG), 6-Phospho-d-glucono-1,5-lactone (6PGL).

Through global analysis of the transcriptomics, proteomics, and metabolomics profiling of CPB cells infected with ISKNV, changes involved in the glucose-related metabolic pathways at 24 and 72 hpi are shown in Figure 1. At 24 hpi, the main alteration found that some enzymes and metabolites of PPP and TCA cycle pathway were upregulated in the mRNA level (6-phosphogluconate dehydrogenase (PGD), Isocitrate dehydrogenase 3 (IDH3), succinate dehydrogenase (SDH)), protein level (G6PDH, Fumarate hydratase (FH)), and metabolic level (Ribose-5-P, histidine, succinate, glutamine). At 72 hpi, compared to 24 hpi, the significant changes showed that some enzymes and metabolites of glycolysis pathway were upregulated in the mRNA levels (Fructose-1,6-bisphosphatase (F16P), fructose-bisphosphate aldolase (ALDO), Glyceraldehyde-3-phosphate dehydrogenase (G3P2), 2,3-bisphosphoglycerate-dependent phosphoglycerate mutase (PGAM2)), protein level (GPI, ENO and Pyruvate dehydrogenase phosphatase (PDP)), and metabolic level (glucose). These results indicated that the ISKNV infection enhanced the metabolic pathways of PPP and TCA cycle at the early stage of the ISKNV infection cycle, and enhanced the glycolysis pathway at the late stage of the ISKNV infection cycle.

**Figure 2:**
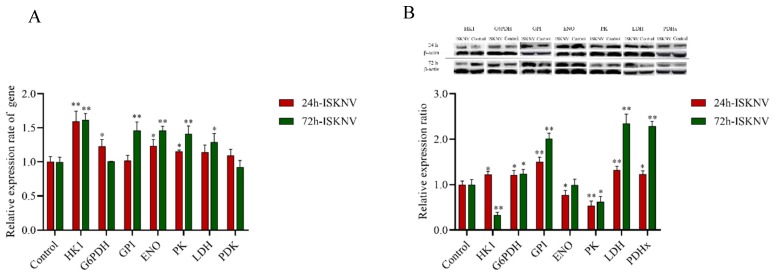
The ISKNV infection altered the glucose metabolism in CPB cells. (**A**) The ISKNV infection promoted the transcription of relative genes of glucose metabolism. (**B**) Western blot analysis of relative genes of glucose metabolism expression in control- and ISKNV-infected CPB cells. At the indicated time points after the ISKNV infection, total protein was extracted from CPB cells and subjected to Western blotting. β-Actin was used as an internal control. For each target protein, three to six parallel samples were pooled as biological replicates. * *p* < 0.05; ** *p* < 0.01.

To further precisely determine the changes of major enzymes involved in glucose metabolism, the expression of seven enzymes were measured by qRT-RCR and Western-blotting. As shown in Figure 2, at 24 hpi, except GPI, PDK, and LDH, the mRNA expressions of five genes were upregulated, including HK1, G6PDH, ENO, and PK. At the same time, there was also a corresponding increase in the protein levels of five enzymes, including HK 1, G6PDH, GPI, LDH, and PDHx, at 24 hpi. At 72 hpi, except G6PDH and PDK, the mRNA expressions of five genes including HK1, GPI, ENO, PK, and LDH were upregulated. At the same time, there was also a corresponding increase in the protein levels of four enzymes, including G6PDH, GPI, LDH, and PDHx, at 72 hpi. These results revealed the similar expression tendency as the transcriptomics and proteomics data, despite some quantitative differences at the expression level. Taken together, the results indicated that the ISKNV infection enhanced the metabolic pathways of the PPP and TCA cycle at the early stage of the ISKNV infection cycle, and the glycolysis pathway at the late stage of ISKNV infection cycle.

### 3.2. ISKNV Induced the Warburg Effect in CPB Cells at the Late Replication Stage

**Figure 3:**
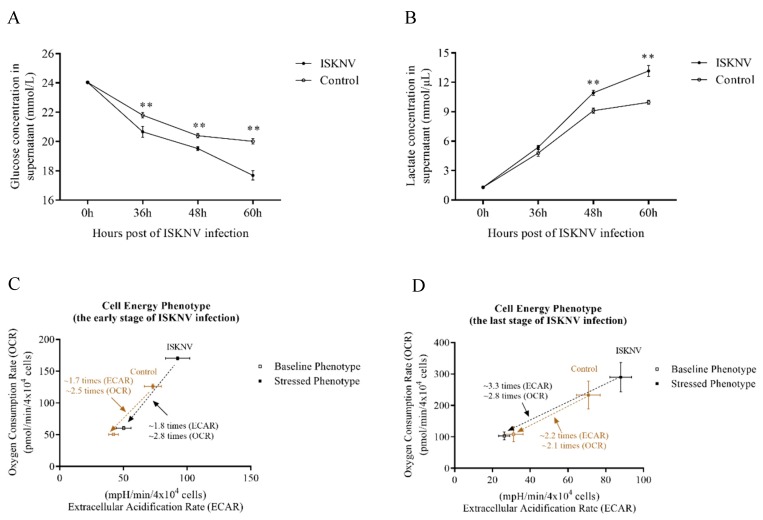
The ISKNV infection induced the Warburg effect at the late stage, but not at the early stage. (**A**) and (**B**) represent the change of concentration of glucose and lactate in supernatant, respectively. (**C**) Seahorse Bioscience extracellular flux analyzer was used to measure the cell energy phenotype (early stage of ISKNV infection) in control- and ISKNV-infected CPB cells. (**D**) Seahorse Bioscience extracellular flux analyzer was used to measure the cell energy phenotype (late stage of ISKNV infection) in control- and ISKNV-infected CPB cells. Data are normalized to cells number. * *p* < 0.05; ** *p* < 0.01.

Our results showed that the ISKNV infection enhanced glycolysis at the late stage of its infection cycle, and the phenomenon was associated with Warburg effect-like metabolic changes. This prompted us to investigate whether ISKNV truly induced the Warburg effect in CPB cells. At first, we examined the effect of the ISKNV infection on glucose uptake. As shown in Figure 3A, the concentration of glucose in the supernatant of ISKNV-infected cells was significantly lower than in control cells, indicating that ISKNV increased the absorption and utilization of glucose during its infection cycle. Then, to determine whether the increased glucose uptake leads to increased aerobic glycolysis, we examined the effect of the ISKNV infection on the production of lactate. As shown in Figure 3B, the production of lactate in supernatant of ISKNV-infected cells was significantly higher than control cells at 48 and 60 hpi, indicating that the ISKNV infection accelerated lactate production in CPB cells. At last, the cell energy phenotype in ISKNV-infected cells was measured using the Seahorse XFp extracellular flux analyzer. As shown in Figure 3C, the cell energetic shift at the early stage of ISKNV infection (36 hpi) showed that ISKNV-infected cells have a ~1.8-times increase in acidification rate and a ~2.8-times increase in oxygen consumption, while control cells have a ~1.7-times increase in acidification rate and a ~2.5-times increase in oxygen consumption. At the late stage of the ISKNV infection (60 hpi), we observed that ISKNV-infected cells have a ~3.3-times increase in acidification rate and a ~2.8-times increase in oxygen consumption, while control cells have a ~2.2-times increase in acidification rate and a ~2.1-times increase in oxygen consumption (Figure 3D). As expected, ISKNV-infected cells mainly increase acidification rate at the late stage of the ISKNV infection. These results implied that the ISKNV infection induced aerobic glycolysis in CPB cells at the late stage of its infection cycle.

### 3.3. Aerobic Glycolysis Was Required for Efficient ISKNV Multiplication

**Figure 4:**
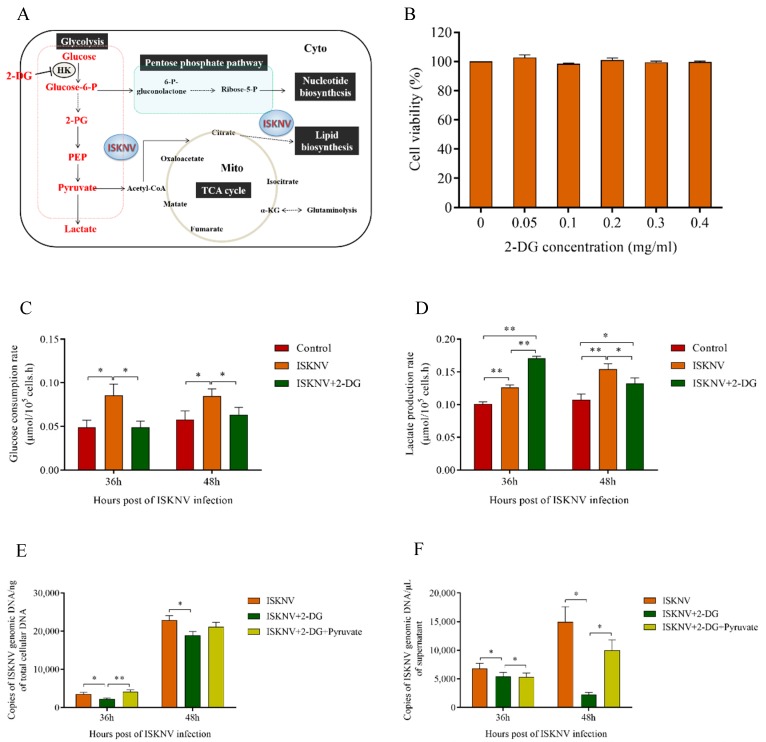
Aerobic glycolysis was required for efficient ISKNV multiplication. (**A**) Overview of cellular metabolism with glucose. 2-Deoxy-d-glucose (2-DG) inhibited glycolysis by inhibiting the expression of HK. (**B**) CPB cells were cultured in complete medium treated with 0–0.4 mg/mL of 2-DG, and the cell viability was detected by MTS assay at 72 hpi. (**C**,**D**) The glucose consumption and lactate production in ISKNV infected CPB cells. (**E**,**F**) CPB cells treated with 2-DG, 2-DG replenished with pyruvate, or without 2-DG were infected with ISKNV. Viral yield in cells and in supernatant was determined by real time PCR. * *p* < 0.05; ** *p* < 0.01.

The results of aerobic glycolysis pathway inhibited with 0.2 mg/mL 2-DG are shown in Figure 4. First, we found that 2-DG inhibited glucose utilization rate (Figure 4C), but interestingly increased the lactate production rate at 36 hpi and inhibited the lactate production rate at 48 hpi in ISKNV-infected cells (Figure 4D). We hypothesized that under the lack of glucose, the glycolysis was replenished rapidly by the other metabolic pathways at 36 hpi, leading to the rapid lactate accumulation.However, the lactate production rate was decreased at 48 hpi with substitutes consuming, which indicated that aerobic glycolysis was important and required for ISKNV multiplication. Then, we explored what happens to ISKNV DNA genome replication when glycolysis is suppressed by inhibition of 2-DG. The results showed that when cells were treated by 2-DG, the copy number of ISKNV genome in cells was reduced by 36.8% and 17.3% at 36 hpi and 48 hpi (Figure 4E), and the number of mature virus particles in supernatant at 36 hpi was reduced by 20.6% (Figure 4F). Particularly, the number of mature virus particles in the supernatant at 48 hpi was significantly reduced by 85% when 2-DG was used to inhibit glycolysis (Figure 4F). The change trend of ISKNV quantity in the supernatant was similar to the lactate production rate, which proved that aerobic glycolysis was important for ISKNV maturation. Finally, when pyruvate was supplemented, the copy number of ISKNV genome was recovered in CPB cells (Figure 4E) and the number of mature virus particles was recovered to 67% in supernatant at 48 hpi (Figure 4F). Taken together, these results indicated that aerobic glycolysis was required for ISKNV multiplication and exogenous pyruvate partly rescued ISKNV production under inhibition of glycolysis.

### 3.4. The Pentose Phosphate Pathway Was Required for ISKNV Multiplication to Provide Nucleotide Synthesis

**Figure 5:**
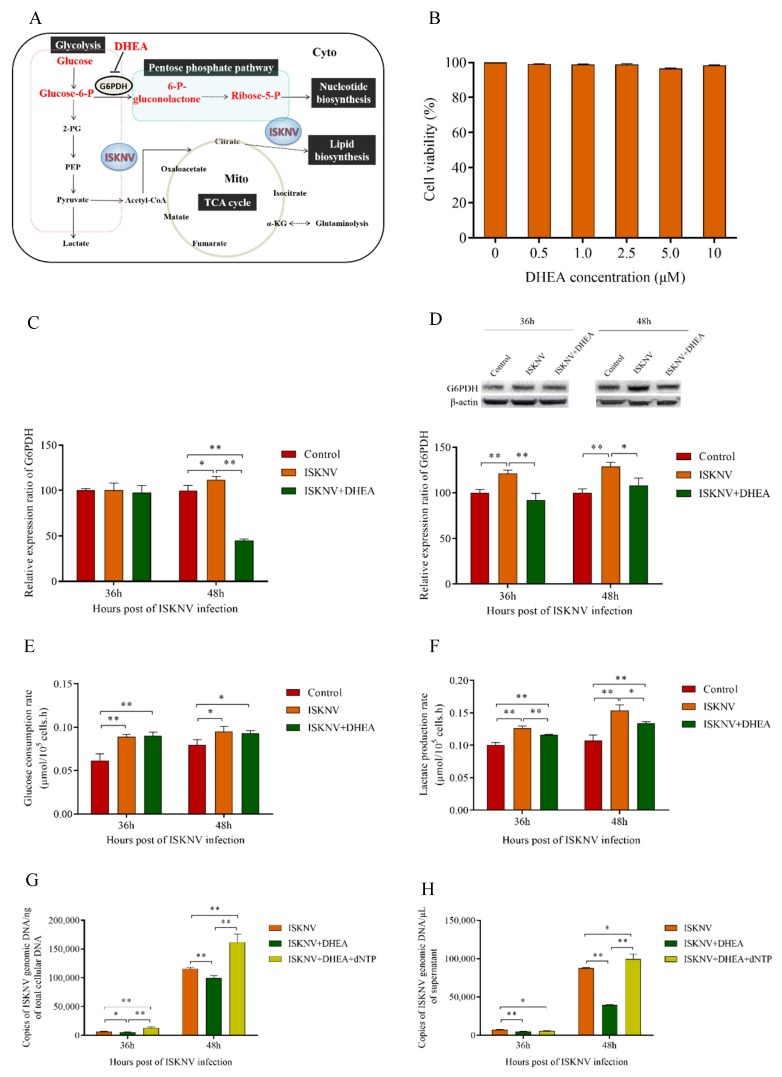
The pentose phosphate pathway (PPP) was required for ISKNV multiplication to provide nucleotide synthesis. (**A**) Overview of cellular metabolism with glucose. Dehydroepiandrosterone (DHEA) inhibited the expression of G6PDH to inhibit Glucose-6-P into PPP. (**B**) CPB cells were cultured in complete medium treated with 0–10 μM of DHEA, and the cell viability was detected by MTS assay at 72 hpi. (**C**) Real-time quantitation of G6PDH expression in CPB cells. (**D**) Western blot analysis of G6PDH expression in CPB cells. (**E**,**F**) The glucose consumption and lactate production in ISKNV infected CPB cells. (**G**,**H**) CPB cells treated with DHEA, DHEA replenished with dNTPs, or without DHEA were infected with ISKNV. Viral yield in cells and in supernatant was determined by real-time PCR. * *p* < 0.05; ** *p* < 0.01.

The results of PPP inhibited with 2.5 μM DHEA are shown in Figure 5. First, we found that DHEA inhibited the gene and protein expression level of G6PDH in ISKNV-infected cells (Figure 5C,D). Next, we detected the glucose consumption rate and lactate production rate of ISKNV-infected cells treated by DHEA. The results showed that there was not a significant difference in the glucose consumption rate between the ISKNV group and ISKNV+DHEA group (Figure 5E), which proved that DHEA did not affect ISKNV to uptake glucose. However, the additional DHEA reduced the lactate production rate in ISKNV-infected cells (Figure 5F). We inferred that the partial glucose entered into other pathways to replenish the PPP. Then, we detected the multiplication of ISKNV in CPB cells treated by DHEA. Figure 5G showed that the copy number of ISKNV genome was reduced by 10.7% and 13.8% at 36 hpi and 48 hpi when cells were treated by DHEA. In particular, the number of mature virus particles in the supernatant was significantly reduced by 30.3% and 54.9% at 36 hpi and 48 hpi when using DHEA to inhibit PPP (Figure 5H). Finally, when dNTPs were supplemented, the copy number of ISKNV genome in CPB cells was significantly recovered to 203.6% and 140% at 36 hpi and 48 hpi (Figure 5G), and the number of mature virus particles in supernatant was recovered to 78.3% and 113.5% at 36 hpi and 48 hpi (Figure 5H).

### 3.5. TCA Cycle Was Not Necessary for ISKNV Multiplication

**Figure 6:**
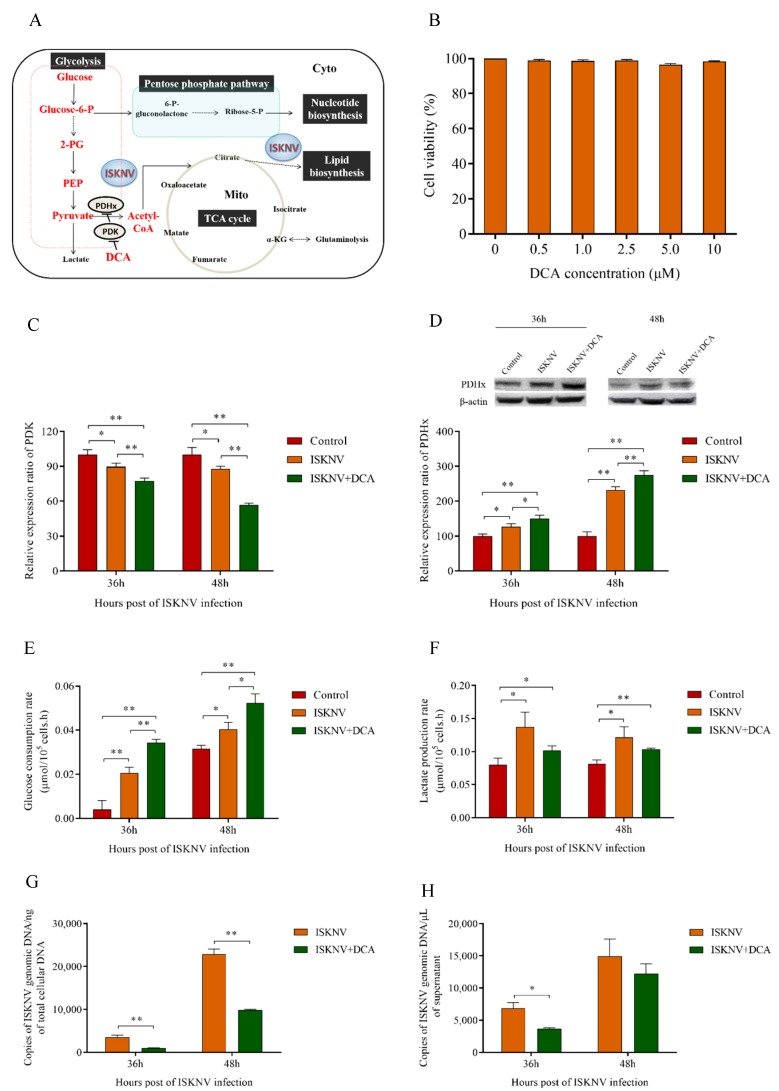
The tricarboxylic acid (TCA) cycle was not necessary for ISKNV multiplication. (**A**) Overview of cellular metabolism with glucose. DCA inhibited the expression of PDK to promote the expression of PDH, which promoted pyruvate into the TCA cycle. (**B**) CPB cells were cultured in a complete medium treated with 0–10 μM of DCA, and the cell viability was detected by MTS assay at 72 hpi. (**C**) Real-time quantitation of PDK expression in CPB cells. (**D**) Western blot analysis of PDHx expression in CPB cells. (**E**,**F**) The glucose consumption and lactate production in ISKNV infected CPB cells. (**G**,**H**) CPB cells treated with DCA, or without DCA were infected with ISKNV. Viral yield in cells and in supernatant was determined by real-time PCR. * *p* < 0.05; ** *p* < 0.01.

As shown in Figure 6, pyruvate was promoted into the TCA cycle with 5 μM DCA. First, we found that DCA inhibited the transcription of PDK and enhanced PDHx expression in the protein levels of ISKNV-infected cells (Figure 6C,D), which indicated that the TCA cycle was promoted. Then, we detected the glucose consumption rate and lactate production rate of ISKNV-infected cells treated by DCA. The results showed that DCA enhanced the glucose utilization rate (Figure 6E) but inhibited lactate production rate in ISKNV-infected cells (Figure 6F), which further proved that DCA promoted pyruvate by entering into the TCA cycle. Finally, we detected the multiplication of ISKNV in CPB cells treated by DCA. The results showed that the copy number of the ISKNV genome in cells and of mature virus particles in the supernatant decreased at 36 hpi and 48 hpi when cells were treated with DCA, in which the copy number of ISKNV genome in cells was significantly reduced by 57% at 48 hpi (Figure 6G,H). These results illustrated that promoting the TCA cycle decreased ISKNV multiplication, and further indicated that the TCA cycle was not important or necessary for ISKNV multiplication.

## 4. Discussion

Viruses depend on host cellular metabolism to satisfy their efficient replication needs. Many viral diseases have been linked to metabolic disorders or metabolic reprogramming. Glucose is one of the most important carbon sources and nutrients, which is required for the synthesis of other amino acids, lipids, and proteins. Glucose metabolism plays an important role in cell metabolism, and it is usually involved in the glycolysis, PPP, and TCA cycle pathways. Recently, the Warburg effect was investigated for some viruses, including HPV [4], HCMV [5,6], WSSV [7,8], KSHV [9], and HCV [10]. Some studies also showed that the infection of some viruses promotes glucose entry to the PPP, including WSSV [8] and HIV-1 [23]. In this study, our results showed that the ISKNV infection induced the Warburg effect at the late stage of the ISKNV infection cycle (72 hpi) in CPB cells, and the accelerated metabolite levels of glycolysis and PPP were required for efficient replication of ISKNV in CPB cells. In addition, TCA cycle was not necessary for ISKNV multiplication. Taken together, these results provide new insights into viral pathogenesis targeting by metabolic shift, as well as antiviral treatment strategies of ISKNV.

The Warburg effect is a metabolic shift that was first found in cancer cells, but was recently also discovered in cells infected by viruses [4,5,7,9,24,25,26,27,28,29,30,31,32]. The first step of the glycolysis pathway is the conversion of glucose to glucose-6-phosphate (G6P) by HK. It prevents the elevated levels of G6P from causing feedback inhibition and thus allows the aerobic glycolysis to continue, even in the presence of sufficient energy and oxygen [33]. LDH produces lactate from pyruvate in the last step of anaerobic glycolysis. It is reported that the increased enzyme activity of LDH was detected during the WSSV infection [34]. Our previous study found that the first infection cycle of ISKNV started from 0 hpi to 72 hpi, and according to the replication kinetics of ISKNV in CPB cells, 24 hpi and 72 hpi were regarded as the early and the late stage of ISKNV replication, respectively [12]. In the present study, ISKNV promoted the expression of HK1 and LDH both at 24 hpi and 72 hpi, which inferred that glucose was being rapidly converted into glycolytic intermediates. Then, we further monitored the glucose consumption and lactate production of CPB cells during an infection cycle of ISKNV and found that ISKNV promoted both glucose uptake and lactate production at 72 hpi. In addition, the cell energy phenotype results also showed that ISKNV-infected cells mainly increased the acidification rate at 72 hpi, suggesting that ISKNV induced aerobic glycolysis in CPB cells at its late stage of infection. The above results proved that the ISKNV infection induced the Warburg effect at the late stage, but not at the early stage. The Warburg effect is characterized by rapid energy ATP generation [2,3]. ATP is an energy source for the binding, maturation, assembly, and budding processes of many enveloped viruses [35]. Our previous study showed that the ISKNV infection induced glutaminolysis to accommodate the biosynthetic and energy needs for its efficient virus replication [17]. We also reported that ISKNV-infected cells contained about 150% more ATP than mock-infected cells at 24 hpi, but ATP level in ISKNV-infected cells was lower by 50% compared to mock-infected cells at 48 hpi, which indicated that glutamine-derived ATP was used for ISKNV replication at the early stage of ISKNV infection cycle [17]. Chambers et al. [36] reported that during the course of HCMV infection, the infected cells became dependent upon glutamine for ATP production, and the addition of extra glucose to the glutamine-free medium did not rescue ATP production. They also found that HCMV induced the switching of anaplerotic substrates from glucose to glutamine within the first 24 h of infection, and this increased significantly by 48 hpi. Thus, we inferred that ISKNV infection induced the Warburg effect at the late stage and mainly supplied ATP for ISKNV maturation. It also has been reported that WSSV infection induced the Warburg effect at the viral genome replication stage (12 hpi) [7]. We presumed that shrimp is a kind of low-grade invertebrate with the incomplete immune system, which may cause the difference in results between shrimp and other vertebrate.

The PPP is one of the important pathways of glucose metabolism, which converts G6P to 6-phosphate-gluconolactone catalyzed by G6PDH for nucleotide biosynthesis. In the present study, the level of glucose was decreased and the activity of G6PDH was increased at the early stage of ISKNV infection (Figure 1), which suggested that the glucose metabolism was being rerouted into the PPP. In cancer cells, the PPP is usually upregulated to balance cellular redox conditions and to ensure an adequate supply of ribose-5-phosphate (R5P) for nucleotide synthesis, a rate-limiting step in cancer cell proliferation [37,38]. Similarly, viral replication requires a great number of DNA precursors supplied by host cell metabolism through salvage reactions or de novo synthesis [39,40]. To ensure an adequate supply of nucleotides, viruses use several strategies, including expressing virus-encoded enzymes that boost nucleotide synthesis and increasing the flux of the PPP [6,10,41,42,43,44]. In this study, we found that ISKNV replication increased the expression of G6PDH, and ISKNV production was rescued by exogenous dNTPs under the inhibition of PPP. These results provided further evidence that ISKNV does, in fact, induce upregulation of the PPP, and PPP strategy providing nucleotide synthesis was required for ISKNV replication. Taken together, these results indicated that PPP was required for ISKNV multiplication to provide nucleotide synthesis.

The TCA cycle, providing precursors of macromolecules in growing cancer cells, is an aerobic respiratory pathway in animal cells in which glucose is converted into ATP and NADPH [11]. Under normal aerobic conditions, cells utilize aerobic respiration and pyruvate catalyzed by PDHx converted to Acetyl-CoA entering into TCA cycle. But under hypoxia conditions, pyruvate converts to lactate catalyzed by LDH. PDK, a kind of kinase, can inhibit the activity of PDHx through phosphorylation. Inhibition of PDK increases the activity of PDHx and promotes pyruvate to enter the TCA cycle to produce energy or macromolecules [45,46,47]. DCA is a small molecular inhibitor that inhibits the activity of PDK and negatively regulates PDHx [48]. In this study, our results showed that DCA inhibited the transcription of PDK and enhanced the expression of PDHx protein, which promoted pyruvate into the TCA cycle. This metabolic shift, induced by DCA, leads to the significant reduction of ISKNV yield. We previously reported that glutamine served as an anaplerotic substrate, replenishing the TCA cycle intermediates and producing ATP via glutaminolysis for ISKNV multiplication in CPB cells [17]. Thus, the results proved that glucose involved in the TCA cycle was not necessary for ISKNV multiplication.

Taken together, our results clearly indicate that the ISKNV infection promotes the involvement of glucose in the Warburg effect and PPP, which is required for ISKNV multiplication.

## 5. Conclusions

In the present study, ISKNV-induced glucose-related metabolic pathways were comprehensively analyzed using the global transcriptomics, proteomics, and metabolomics profiling. The Warburg effect, PPP, and TCA cycle pathways were detected and analyzed. The analysis results suggest that ISKNV replications induce the Warburg effect in CPB cells at the late replication stage, which is required for its multiplication. The PPP is also induced for nucleotide synthesis to promote ISKNV replication. However, the TCA cycle involving glucose is not necessary for ISKNV multiplication. The present study may be a foundation for further research and can provide new insights for viral pathogenesis mechanism of metabolic shift. The pathogenesis mechanism will be confirmed by further studies.

## Figures and Tables

**Table 1:** Primers used in this study.

Primer Names	Gens Name	Sequence (5′-3′)
q-18s-F	18S rRNA	CATTCGTATTGTGCCGCTAGA
q-18s-R		CAAATGCTTTCGCTTTGGTC
q-HK1-F	Hexokinase 1 (HK 1)	TTATCCGTCCCTCAAATAGCA
q-HK1-R		GGCTCTATCAACCCAGGAAAG
q-GPI-F	Glucose-6-phosphate isomerase (GPI)	CTCTGGTCGCCATGTATGAG
q-GPI-R		CTCCGGCTCGATCTTCTTC
q-G6PDH-F	Glucose-6-phosphate dehydrogenase (G6PDH)	ACCGCTCTGCTTCTGTATCC
q-G6PDH-R		CTTTGCTCGCTCTGACTTGA
q-ENO-F	Enolase (ENO)	TGCACTGGACAGATCAAGACA
q-ENO-R		AGCTCCTCTTCAATCCTGAGC
q-PK-F	Pyruvate kinase (PK)	GATGAAGGAGGCAAAGACCA
q-PK-R		GCAGCAAGAAGGGAGTGAAC
q-LDH-F	Lactate dehydrogenase (LDH)	GGACAGTGCCTACGAGGTGA
q-LDH-R		GGTAGAGACAGGATGGACAC
q-PDH-F	Pyruvate dehydrogenase (PDH)	CGTTGTGCCTGTTTCTGATG
q-PDH-R		CAAATGGTGCAGAGCTGGTA
